# Medical Double Talents: How Medical Students Living with Chronic Conditions Teach Their Peers

**DOI:** 10.5334/pme.1839

**Published:** 2025-10-09

**Authors:** Casper G. Schoemaker, Sanea Smits, Elsemarijn L. Leijenaar, Maaike Kok, Megan Milota

**Affiliations:** 1 University Medical Center Utrecht, Utrecht, The Netherlands; 2 Department of Bioethics and Health Humanities, University Medical Center Utrecht, Utrecht University, Utrecht, The Netherlands

## Abstract

**Background & Need for Innovation:**

Approximately 10% of medical students live with a chronic somatic or mental condition. They often feel alienated in the dominant culture of invulnerability in medical education. We developed the ‘Medical Double Talents’ (MDT) innovation to facilitate medical students living with chronic conditions – MDTs – to teach their fellow medical students.

**Goal of Innovation:**

The goals of the MDT sessions are to increase medical students’ empathy and compassion for patients with chronic conditions, to challenge the implicit culture of invulnerability, and to help MDTs turn their patient experiences into a strength.

**Steps taken for Development and Implementation of innovation:**

The MDT sessions were developed using a co-design approach, engaging medical students with chronic conditions at high levels of patient involvement. These students built a supportive community, received training, and shared their unique perspectives during teacher-facilitated guest lectures and small group discussions.

**Evaluation of Innovation:**

In November 2024, 263 medical students participated in 24 MDT sessions. They reported highly appreciating the personal insights shared. The MDTs felt empowered by the experience as lecturers. The student-led dialogue enhanced engagement, though some students desired more information about the medical context before the session. These sessions complement traditional training.

**Critical Reflection on Your Process:**

The MDT sessions challenge the implicit culture of invulnerability in medical education. The shared experiences can help foster empathy, self-reflection, and transformation in their peers. This innovation, integrated into Utrecht’s curriculum, highlights the need for safe learning spaces and community building to implement similar programs elsewhere.

## Background & Need for Innovation

Active patient involvement in medical education helps students develop new knowledge, skills, and attitudes contributing to person-centeredness; it also makes education more engaging, significant, and transformative [1,2]. Moreover, the active involvement of patients improves empathy and compassion for living with chronic illness in the real world [3,4,5].

Typically, patients involved in medical education are recruited from outside the medical school. This unintentionally confirms the incorrect assumption that medical students and lecturers cannot be patients. Approximately 10% of medical students live with a chronic somatic or mental condition [6,7,8]. They face pervasive social, cultural, and structural barriers to accessing and excelling in educational environments (e.g., social stigma and fear of downstream consequences of disclosure, absent or burdensome accommodation processes, and inflexible curricula and training programs) [6,7,8].

These students constitute a hidden minority in medical education, as they are reluctant to disclose their condition to peers or faculty [9]. They often feel alienated in the dominant culture of invulnerability [10]. For example, ‘textbooks, lectures, and PowerPoints speak about patients as if they are other people’ [7]. In this learning environment, the concept of a physician with a chronic condition is perceived as inappropriate for the profession [7,8].

Stergiopoulos et al. use the term ‘identity compartmentalization’ to describe how these students learn to develop a dual identity [8]. During medical education, they are ‘healthy’ invulnerable medical students, and at home, they are vulnerable ‘sick’ patients [8]. These separate identities impose starkly conflicting demands [6]. The ‘good student’ is expected to self-sacrifice in the service of academic performance, while the ‘good patient’ is expected to self-manage and prioritize their health. It is nearly impossible to perform to the perceived expectations of both roles simultaneously [8].

Stergiopoulos et al. use the term ‘identity intersection’ to describe how medical students with chronic illness could bring together these two identities and use their patient role as an asset rather than a liability [8,9,11]. Thus far, most medical curricula fail to provide the space for students to express their experiences as patients [9,12]. This is a missed opportunity, as their experiences in both roles could improve their fellow students’ communication, empathy, and compassion and change medical school culture [7,10,11,13].

This paper describes the development and evaluation of the “Medical Double Talents” (MDT) program for the medical school at the University Medical Center Utrecht (UMCU), in the Netherlands [14]. In this program, medical students living with chronic conditions – MDTs – are trained to teach their fellow medical students in small-group, facilitated lecture and discussion sessions. These MDT sessions have been integrated into the formal curriculum.

## Goal of Innovation

The primary goal of the MDT sessions is to increase medical students’ empathy and compassion for patients with chronic conditions. The second goal is to break the implicit culture of invulnerability in medical school and promote vulnerability in the learning environment. The third goal is to help MDTs learn to turn their patient experiences into a strength or talent.

## Steps taken for Development and Implementation of innovation

### Co-design approach

We developed the MDT program and the MDT sessions through a co-design approach [15]. Medical students living with chronic conditions were involved in designing the session format and learning objectives and had a high level of autonomy during their lectures [14]. The innovation is a practical example of the UMCU’s commitment to patient involvement [1,5]. The MDTs’ involvement corresponds to the highest levels of patient involvement in medical education [2].

### Community building and identity development

Community building to support the identity development of MDTs was at the heart of the development of the innovation. Most medical students living with chronic conditions are not ready to disclose and discuss their situation in education [7,8,12,16]. Before they can give an MDT guest lecture, they require a safe environment to meet others and experience a reflexive and social learning process together to gradually integrate their two separate identities [8].

Inspired by Forber-Pratt and Zape’s four statuses of identity development in college students with disabilities, we took the following phases of identity development of MDTs into account: 1) *acceptance* of the condition, 2) *relationship* with other MDTs, 3) *adoption* of the concept of MDT, and 4) *engagement* in education as a MDT [16].

We used many channels to call on medical students with chronic conditions to get involved. About 30 students – mainly in the *acceptance* phase – responded. They had an introductory (online) meeting with the coordinator (CS). Most students did not know other medical students with chronic conditions. They were invited to meet each other in quarterly ‘MDT Cafés’ held at the Utrecht Medical Student Association building, a safe and supportive space where they could informally share their stories and build a community [7]. Doctors with chronic conditions were invited as guest speakers and role models [10,17]. The hallmark of this *relationship* phase is the network one builds by meeting other MDTs and forming relationships to learn the ways of the group. The *adoption* phase is an intermediary between simply meeting with other MDTs and engaging in education [16].

Members of our MDT community were asked to give guest lectures, but only if they felt adequately prepared and comfortable. If needed, they first attended a lecture of another MDT. In this *engagement* status, they became role models to new MDT members and gave guest lectures or advocated for curriculum development. This phase is about identity synthesis and embracing one’s experiences as a strength [12,16].

### Multiple personal perspectives

For the development of the sessions, we followed tips for patient involvement in medical education [1,2,3,5], such as fostering a culture of openness, small group discussions, institutional support, rewards for patients, and support for patients, students, and teachers. We explained the MDTs to convey their personal perspectives; they are not expected to bring a collective perspective on behalf of all patients with the same condition [1]. Many MDTs do not know other patients with the same condition.

However, the MDTs can describe more than just the patient’s perspective; they are also medical students and future doctors [1]. For that reason, the MDTs could be considered an embodiment of Akkerman and Bakker’s boundaries in education [18]. As the authors describe it, ‘the boundary belongs to both one world and another. It is precisely this feature that seems to explain how the boundary divides as well as connects sides’ [18]. Positioned at the boundary between patient and healthcare provider, the MDTs’ multiple perspectives carry powerful learning potential to break the implicit culture of invulnerability [11].

We built on diverse bodies of knowledge from domains such as patient involvement in medical education, disability studies, ableism, hidden curriculum, social identity complexity, professional and personal identity formation, adversarial growth, perspective-taking, empathy, boundary crossing, near-peer teaching, role modeling, diversity, and inclusivity. These theoretical backgrounds share a common goal of narrowing the gap between doctors and patients by breaking the implicit image of the invulnerable medical professional. In their vulnerability, MDTs are regarded as courageous disrupters of a dominant culture [11,12].

However, major differences in terminology and definitions exist between these bodies of knowledge. International literature usually refers to ‘medical students with disabilities’. We deliberately narrowed our scope to ‘medical students living with a chronic condition’. This narrower definition of MDTs ties in directly with the content of the communication lessons in medical school: the conversation between doctor and patient. The MDTs are both doctors and patients. They can play an invaluable role in bringing these worlds together [18]. We emphasize that the MDTs are not meant to replace patients from outside the medical school. Hopefully, they will increase students’ receptivity to these patients’ stories.

### Implementation

In March 2023, the communication teachers at the UMC Utrecht invited the MDTs to conduct 30-minute sessions to 24 small groups of second-year bachelor’s medical students during November 2023. These sessions were part of 3-hour mandatory workshops on communication with chronically ill patients. The sessions were given again in November 2024.

At the start of the MDT session, the teacher emphasized confidentiality. In a 5-minute personal introduction (‘guest lecture’), the MDTs then discussed their experience with the medical program, their chronic condition and treatment history, the consequences for their daily life and studies (including internships), their experiences in dealing with doctors (including positive and negative role models), and their career prospects. After the talk, the remaining 25 minutes were devoted to informal discussion. The students were invited to ask questions, which determined the direction of the discussion. The communication teacher assisted the discussion, if necessary, for example by asking follow-up questions or introducing a new discussion topic.

The MDTs received training to practice telling their story and answering questions. The communication teachers were briefed before the session. They received a teacher guide with information about the intervention structure, learning objectives, and their role in preparing the students. The teachers introduced the MDT session during the preceding class and encouraged students to prepare general questions for the MDT in preparation for the discussion [19]. The students in the groups did not know the MDTs’ condition before the start of the session. The teachers were aware that students in their group may decide to disclose their condition for the first time, before or after the MDT session. After the session, the teachers facilitated reflection and aftercare in their groups. The MDT coordinators (CS and NK) facilitated reflection and aftercare for the MDTs.

## Evaluation of Innovation

In November 2024, MDT sessions were given in 24 groups with a maximum of 13 second-year bachelor medical students. In total, 263 students attended these sessions given by eight MDTs and facilitated by 15 communication teachers. The Dutch Association for Medical Education (NVMO), ERB file number 2024.5.2, provided ethical approval for an evaluation study.

After the lectures, 235 students (89% response) anonymously completed a short questionnaire consisting of 12 Likert scale questions: strongly disagree – strongly agree [1,2,3,4,5], one question about the length of the session (too short, right, too long), and three open-ended questions. See Table 1 for the results of the closed questions.

**Table 1:** Students’ scores on closed questions – Likert scale 1 (strongly disagree) – 5 (strongly agree) – in a short survey after the MDT sessions.


Nr	QUESTION	M	SD	RANGE	MEDIAN	MODUS	% AGREEMENT

	*Appreciation for the MDT session*						

1	I find it instructive to hear the story of the MDT.	4,70	0.48	3–5	5	5	99%

2	I could empathize with the story of the MDT.	4,14	0.68	2–5	4	4	86%

3	It has added value that the MDT is our age and is also studying medicine.	4,42	0.66	2–5	5	5	91%

4	I want another guest lecture from an MDT.	4,24	0.82	2–5	4	5	81%

	*Preparation of the MDT session*						

5	I was adequately prepared for this *MDT session*	3,58	0.85	1–5	4	4	59%

6	I would have liked to know in advance the condition it would be about.	3,03	1.07	1–5	3	2	36%

	*Conversation with the MDT*						

7	I like that we were allowed to shape the conversation with the MDT.	4,36	0.63	2–5	4	4	93%

8	I felt free to ask anything I wanted to ask.	4,34	0.71	2–5	4	4	90%

	*Expected impact of the MDT session*						

9	My perception of life with a chronic condition has changed.	3,41	0.90	1–5	3	4	49%

10	Thanks to this class, I can communicate better with patients with chronic conditions.	3,50	0.74	2–5	4	4	52%

11	Thanks to this MDT session, I will become a better doctor.	3,75	0.70	1–5	4	4	70%

	*Connection to the theme of diversity and inclusiveness*						

12	This MDT session fits the theme of diversity and inclusiveness.	3,98	0.80	1–5	4	4	77%


Overall, the students greatly appreciated the MDT sessions. They felt the MDTs’ story was instructive (99% agreement) and appreciated the added value that the MDT was of their age and studied medicine (91%). Most students (59%) felt prepared for the MDT session. Interestingly, 36% would have preferred to know the MDTs’ condition beforehand: 37% did not. The students appreciated the freedom to shape the conversation (93%) and felt free to ask questions (90%). In total, 84% of students felt the length of the session was good; for 15%, it was too short. Half of the students (52%) expected a possible long-term impact on their communication with patients.

After the sessions, focus groups were organized to evaluate the overall experience and session outcomes. Four individual focus groups were held by SS and EL, with students (n = 6), MDTs (n = 5), and communication teachers (n = 2; n = 3). The transcripts of the focus groups and the open-ended questions of the questionnaire were thematically analyzed [20] by SS and EL. They met with CS and MM to discuss preliminary and revised codes. This analysis resulted in four main categories (Table 2).

**Table 2:** Results of the qualitative thematic analysis of focus groups and open questions in the questionnaire: themes and quotes.


LEARNING OUTCOMES STUDENTS	

Recognizing the person behind the patient Understanding that everyone could be a patient Gaining insight into perspectives and perspective-taking Expected improvement of communication and attitude Valuable curriculum addition and preparation for clinical internships Predicted contribution empathetic behavior	1) *“Thanks to this session, I’ve realized that patients are everywhere. People you wouldn’t expect or people with conditions that aren’t visible often keep things to themselves. Once you start paying attention, you begin to notice visible things, like how many people walk with a limp or use a wheelchair. Everyone knows someone [with a condition], but it’s not something people talk about.”* [Student – Focus group] 2) “*You begin to reflect on how a chronic illness, whether you want to or not, can become a defining part of a patient’s identity. As a doctor, it’s essential to distinguish the illness from the person. Additionally, I realized that I had insufficient insight into the profound impact of a chronic illness on a patient, but this lecture has significantly improved my understanding.”* [Student – Questionnaire] 3) *“Apart from the conversation technique, [the MDT session] also influences the group dynamics. Which in turn influences all learning during the communication lesson, because you need that vulnerability to be able to learn about your own communication.”* [Communication teacher – Focus group] 4) “*I think it’s also important to keep in mind when working with real patients, these (MDTs) are also real patients. So to take it with you into the internships and remember: ‘Okay, just treat patients as you would normally treat someone.”* [Student – Focus group] 5) *“And ultimately, if you have that empathy and that idea [of what it is like to be chronically ill], which you learned from those MDTs, then you can incorporate that into your conversations. Which will really improve your conversations and conversation skills.”* [Communication teacher – Focus Group]

**LEARNING OUTCOMES MDTs**	

Empowerment from sharing personal experiences in guest lectures Reflection on own story after sharing this out loud	6) *“And for me, it was also really an ‘eye-opening’ experience that a whole group of people would accept your entire story as the truth. I really didn’t expect to get that out of the session, but I’ve often had moments with doctors where, in the small remarks they make or the questions they ask, you can sense that they doubt whether what I’m saying is true, or if it was really that bad, or if it truly limits me. But these students didn’t question what I was telling them. That really helped me feel empowered in my ‘double role’, because I thought: ‘Hey, the experiences I’ve had are real, and they were tough, and things like that shouldn’t have happened.’”* [MDT – Focus group] 7) *“What I’ve noticed in myself is that sometimes I don’t fully realize – or I try to acknowledge that I have certain limitations. But in my day-to-day activities, I often don’t act like they’re there. So maybe I haven’t accepted it as much as I thought I had. When people ask me, ‘What do you need to keep in mind?’ I find myself thinking: ‘Actually, I don’t always pay attention to this, or that.’ That made me reflect: How well do I take care of myself?’ and ‘When do I allow myself to rest?’ Sometimes I push myself too far because I’m not experiencing symptoms at the moment. And now that I’m talking about this more and people ask questions, it’s made me think even more deeply about it.” [MDT – Focus group]* 8) *“I’ve noticed that my story has changed a bit—not in big ways, but in small things—and that I’m making it more personal because I’ve found that it just has more impact that way. I think that in the beginning, I maybe didn’t quite dare to do that yet. But now I notice that when I do, it actually goes well, and I still feel okay. And that gives it more impact, and I find that valuable somehow—that feeling of: ‘Oh, if I tell it like this, maybe it can reach the students just a little bit more, and help me get across what I really want to share.’” [MDT – Focus group]*

**DYNAMICS LEARNING CLIMATE**	

Inspiring students to share personal experiences Improving group bonds, trust and active engagement in group discussions Appreciation of MDTs Addressing taboos around health conversations	9) “*I had expected that after hearing the story, they would just have a brief discussion and then take a break. I thought I would refer back to what we had learned in my next modules, but instead, immediately a whole group conversation started about the health conditions that students have themselves*.” [Communication teacher – Focus group] 10) “*Everyone shared things afterward, including things that made me think, ‘Oh, I didn’t actually know that about you.’ It really helps you become a bit more open, and you get to know more about each other—things we wouldn’t have known otherwise*.” [Student – Focus group]

**FACILITATORS AND BARRIERS**	

Authentic personal experiences Focus on experiences over students’ performance Relatability of MDTs as peer students The power of MDTs’ vulnerability Student-led dialogue and pre-existing group dynamics Absence of background information MDT-dependent impact of guest lecture Influence educators	11) “*I find it especially useful because they are students our age. This makes the impact of a chronic illness even clearer, as it allows for a better comparison with our own lives*.” [Student – Questionnaire] 12) “*This way, we could discuss experiences of being chronically ill with someone our age and from the same study. That made me feel much more comfortable asking questions, and I also felt that the answers aligned with what I wanted to know*.” [Student – Questionnaire] 13) *“I would have liked to know which illness the person has in advance to prepare more specific questions.”* [Student – Questionnaire] 14) “*I liked that time was spent on other things than just the illness itself*.”’ [Student – Questionnaire]


Students realized that everyone could be a patient and recognized that every patient is an individual with their own life story [Quote 1]. Not only did they gain insight into the daily impact of chronic illnesses, but they also developed a deeper understanding of perspective-taking skills [Quote 2]. Furthermore, teachers mentioned these lessons could contribute to students’ communication skills as students might feel more confident in asking sensitive questions and have learned more about professional attitudes [Quote 3, 4]. Next, teachers described how these sessions could contribute to empathy development [Quote 5]. Teachers highlight the importance of this contribution because students’ empathy generally decreases during internships [4].

The MDTs described feeling empowered after sharing their personal experiences, as they felt heard and acknowledged in their lived experience [Quote 6]. At the same time, sharing their stories stimulated personal reflection on these experiences and their coping [Quote 7]. MDTs described how students’ questions sometimes provided new perspectives on their experience and mentioned how their stories evolved after sharing and discussing them multiple times. [Quote 8] Some students asked questions they had not previously contemplated.

Teachers and students described that the strength and vulnerability displayed by MDTs during the sessions inspired students to feel comfortable sharing their personal (chronic) health conditions with peers [Quote 9, 10]. Teachers also believed that addressing this together and reflecting on strategies for coping with health conditions fostered a group bond and a safe learning climate.

Students and teachers appreciated that the MDTs shared authentic stories focused on experiences, not medical conditions. Students reported they could relate to the MDT and felt more comfortable asking the MDTs questions because they were the same age and fellow students [Quote 11, 12].

In addition, students valued the student-led dialogue and the opportunity to connect medical and theoretical knowledge with real patient contact, which they felt prepared them for clinical practice. Some students reported preferring additional background information on the chronic illness of the MDTs to feel more prepared to ask questions, while most valued that the sessions’ focus was not the illness itself [Quote 13, 14]. Also, some teachers and students expressed that they would have preferred prior notice that some MDTs could have a mental health condition. They felt this could be a particularly sensitive topic for students, especially if someone has personal experience with mental health conditions. At first, some teachers and students assumed every chronic condition to be somatic, which caused a sense of unexpectedness or unpreparedness and a certain level of surprise.

Finally, teachers and MDTs described how all sessions went differently and were unique as MDTs shared their personal stories in several groups in the presence of various teachers.

In anticipation of the next round of MDT sessions in the upcoming academic year, we are currently amending the teacher guide. We are adding a section describing the fact that a chronic condition can be both somatic and mental. We would also like to study the impact of the MDT sessions longitudinally; ideally, we would like to assess how MDTs perceive their role as facilitators over multiple years, and how repeated teaching experiences impact both their professional and personal identity formation. We are also interested to learn what the long-term impacts of the MDT sessions are for medical students. Interviewing participants at regular intervals after the session (for instance once per year), or administering an annual short survey are possible means of capturing relevant data on the lasting impacts of this intervention.

## Critical Reflection on your process

Through this project, we learned a lot about prevailing assumptions in standard medical education. For instance, we encountered the assumption that a chronic condition is somatic, and not mental. This led to some discomfort when MDTs shared their mental condition. In future MDT sessions, teachers and students should be even more explicitly informed about the possibility of somatic and mental conditions.

We also learned that medical education is often strictly divided into diseases and pathologies. If patients are invited to participate in education, students are only asked to research their specific conditions in preparation for the lesson. For the MDT sessions, we purposefully chose a different preparation process to avoid disease-specific, strict medical discussions. Finally, the high degree of freedom to shape the discussion was unusual and experienced as enjoyable by most medical students [19].

Some students and teachers from other medical schools in the Netherlands have expressed interest in these sessions. Our key recommendation for them is to bring together a group of MDTs so they can build a community at their medical school and pinpoint the most appropriate part of their curriculum. We have found it challenging to get these students actively involved. Due to their conditions, most MDT students need their energy for their compulsory programs. Expanding this innovation therefore needs support from teachers and management, financial backing, and innovation expertise.

## Conclusion

The MDT sessions appeared to increase medical students’ empathy and compassion for patients with chronic conditions. The MDTs helped to challenge the implicit culture of invulnerability and promote vulnerability in the learning climate. The MDTs learned to turn their patient experiences into a strength or talent. The MDT sessions have been integrated into the curriculum in Utrecht, the Netherlands. Building a community of MDTs is the first step in introducing this innovation to other medical schools.
